# Longitudinal changes in self-reported medication adherence and beliefs about post-stroke medicines in Sweden: a repeated cross-sectional study

**DOI:** 10.1136/bmjopen-2024-084680

**Published:** 2024-10-18

**Authors:** Maria Sjölander, Maria Gustafsson, Henrik Holmberg, E-L Glader

**Affiliations:** 1 Department of Medical and Translational Biology, Umeå University, Umeå, Sweden; 2 Department of Epidemiology and Global Health, Umeå University, Umeå, Sweden; 3 Department of Public Health and Clinical Medicine, Umeå Universitet, Umeå, Sweden

**Keywords:** Behavior, CLINICAL PHARMACOLOGY, Stroke medicine, PREVENTIVE MEDICINE

## Abstract

**ABSTRACT:**

**Objectives:**

To explore changes in beliefs about medicines and self-reported medication non-adherence between 3 and 24 months after stroke and to investigate associations between beliefs about medicines and non-adherence at 24 months after stroke.

**Design:**

Longitudinal questionnaire survey.

**Setting:**

Patients treated for acute stroke in 25 Swedish hospitals.

**Participants:**

Only patients living at home were included. Of the 594 individuals who answered the 3 month questionnaire, 401 were included at 24 months; among the remainder, 34 (5.7%) had died, 149 (25,1%) did not respond or had incomplete information on adherence and 10 (1.7%) were not living at home.

**Measures:**

The primary outcome was self-reported medication adherence as measured with the Medication Adherence Report Scale (MARS). The Beliefs about Medicines Questionnaires (BMQ) was used to assess personal beliefs about medicines. Background and clinical data were included from the Swedish national stroke register.

**Results:**

According to dichotomised MARS sum scores, more individuals were classified as non-adherent at 24 months after stroke (n=63, 15.7%) than at 3 months after stroke (n=45, 11.2%) (p=0.030). For BMQ, the only difference over time was an increase in the *Necessity* subscale (p=0.007). At 24 months, in comparison to adherent patients, non-adherent patients scored statistically significant higher on negative beliefs about medicines, such as *Concern* (OR 1.17, 95% CI: 1.09 to 1.25), *Overuse* (OR: 1.37, 95% CI: 1.21 to 1.54) and *Harm* (OR: 1.24, 95% CI: 1.11 to 1.39), and lower on positive beliefs about medicines, namely, *Necessity* (OR: 0.88, 95% CI: 0.80 to 0.96) and *Benefit* (OR: 0.85, 95% CI: 0.74 to 0.98).

**Conclusions:**

Stroke patients‘ beliefs about medicines were associated with adherence, and over time beliefs remained stable across all domains, except for an increased perception of medications as being necessary. Despite this, more patients became non-adherent over time. To counteract non-adherence, interventions targeted to improve intentional adherence as well as non-intentional adherence should be investigated and implemented.

**Strengths and limitations of this study**

- A consecutive sample of individuals with stroke, treated in 25 Swedish hospitals covering both rural and urban areas, was included in this questionnaire survey, and the response rate was high.
- This study used previously validated questionnaires to estimate self-reported medication adherence and personal beliefs about medicines.
- The longitudinal follow-up (3 and 24 months after stroke) enabled us to examine changes over time in medication adherence and personal beliefs about medicines.
- Analyses of non-responders showed that those who did not respond were older and in poorer health than those who did respond.
- There are methodological challenges to measuring medication adherence in general and self-reported adherence in particular.

## Introduction

 Stroke is a major cause of death and disability worldwide.^1^ In Sweden, approximately 20 000 new stroke events are annually registered in Riksstroke, the Swedish stroke register with more than 90% coverage of acute stroke patients.^2^ Preventive medications such as antihypertensive, antithrombotic and lipid-lowering drugs are effective in lowering stroke risk and are recommended in guidelines.^3^ In Sweden, most stroke patients are prescribed these preventive medications. However, previous Swedish studies have indicated suboptimal adherence to preventive drug treatment, even in patients who have experienced a stroke. During the first year of treatment, the adherence rates were found to be 85% and 88%, respectively, for antiplatelets and antihypertensives but as low as 67% for statins and 69% for warfarin and with even lower rates after 2 years.^4 5^ As drug adherence improves cardiovascular risk as well as treatment outcome and survival, understanding people’s adherence to drug treatment is crucial for optimal stroke prevention.^6 7^

Non-adherence can be both unintentional, for example, forgetting or misunderstanding the treatment, and intentional, if there is an active decision to not use a medication. The Howard Leventhal’s Common-Sense Model of Self-Regulatory Model is frequently used as framework for the study of medication adherence, which aims to describe and understand the process involved to initiate and maintain behaviour in relation to illness threats.^8 9^ If the patient perceives common sense in relation to beliefs and experiences, when taking the prescribed medication, adherence will be more likely. The Necessity-Concern framework is a potential model for the relationship between beliefs and adherence.^10^ The association between individual beliefs about medications and adherence is also assumed to be stronger for intentional non-adherence as intentional non-adherence involves a decision based on reasons that the individual considers important and relevant.^11^ The Beliefs about Medicines Questionnaire (BMQ) is a validated and well-established instrument, which distinguishes between general beliefs (drug treatment in general) and specific beliefs (the respondent’s own drug treatment).^8^

During the first month after stroke onset, intentional primary non-adherence to preventive drug treatment has been shown to be low and not associated with beliefs about medicines.^12^ Instead, factors related to unintentional non-adherence, such as changes in daily living and a greater need for help, are more important. Non-adherence to drug treatment after stroke has been shown to increase over time, while in some studies, general beliefs about medicines have been shown to remain stable.^4 13^ Previous research has also shown that with an increasing number of medications, the risk of the patient experiencing concerns rises.^14^ Many stroke patients are subject to polypharmacy and stability of beliefs about medicines needs to be explored and in specific in relation to stroke. In our previous study, self-reported non-adherence was associated with general and specific beliefs about medicines at 3 months after stroke, indicating that intentional non-adherence may have a greater impact on drug treatment beyond the first month.^15^ To determine whether to intervene on intentional or non-intentional non-adherence, the relationship between beliefs and adherence in stroke patients needs to be studied for even longer time periods.

This study is an extension of our previous study on self-reported non-adherence and beliefs about medicines at 3 months after stroke onset.^15^ The hypotheses were that beliefs would be stable, non-adherence would increase over time and beliefs about medicines would be associated with non-adherence.

The specific study objectives were:

1. To investigate whether personal beliefs about medicines and self-reported non-adherence to drug treatment changed between 3 and 24 months after stroke onset.
2. To assess whether beliefs about medicines were associated with long-term self-reported non-adherence.

## Methods

This longitudinal repeated cross-sectional questionnaire survey was conducted among stroke patients treated in Swedish hospitals. The study questionnaire consisted of three translated and previously tested questionnaires: the 5-item Medication Adherence Report Scale (MARS),^16^ the Brief Illness Perception Questionnaire (not used in the present analysis)^17^ and the BMQ.^8^ The same questionnaire was answered by the same individuals on two occasions, 3 and 24 months after stroke onset. This study followed the Strengthening the Reporting of Observational Studies in Epidemiology cohort reporting guidelines.^18^

In 2011, all hospitals that treated patients with acute stroke in Sweden participated in Riksstroke, and the coverage in 2011 was estimated at 90.5%. All hospitals were asked to participate in the present study, and 25 of 75 hospitals volunteered. Participating hospitals included university hospitals, large regional hospitals and smaller local hospitals and covered different geographical areas in Sweden, including 15 of Sweden’s 21 counties/regions and both urban and rural areas. Patients who are treated for acute stroke in a Swedish hospital are asked to participate in the register, and only a small proportion decline. Riksstroke includes information during hospital stay on patient background from before the stroke, from the acute care as well as preventive medications and rehabilitation. Riksstroke also includes 3- and 12 month follow-ups, using questionnaires sent to the patients, and this information was not included in this study.

Validations have shown high consistency between Riksstroke and medical reports.^19^ Patients included in this study had a stroke from December 2011 to April 2012 and were treated in one of the participating hospitals. The 3 month study questionnaire including MARS and BMQ was sent by mail together with the usual 3 month follow-up questionnaire from Riksstroke and was answered from March 2012 to June 2012. The 24 month study questionnaire was sent by mail to the individuals who were still alive and was answered from December 2013 to March 2014. Participants who were in an assisted living such as nursing homes 3 and 24 months after onset of stroke were not included in the analyses, because non-individual routines were expected to influence medication adherence.

Data from the questionnaires were linked to data from Riksstroke through the personal identification number possessed by all residents in Sweden.^20^

### Patient and public involvement

Neither patients nor the public were involved in the design, conduct, reporting or dissemination plans of this research.

### Variables

The main outcome was self-reported adherence to drug treatment, measured with MARS.^21 22^ MARS has shown satisfactory validity (0.7 correlation with adherence) and reliability (0.67–0.89 Cronbach’s alpha) when translated into other languages and is used for several illnesses.^16 23 24^ MARS consists of five Likert-style questions on how prescribed drugs are used, and respondents answer on a 5° scale (never=5, seldom=4, sometimes=3, often=2, always=1) regarding how often they engage in different types of non-adherent behaviours. A sum score was calculated with minimum 5 and maximum 25, with higher scores indicating greater adherence. A missing answer on any of the questions resulted in a missing answer on the total score.

Patients’ beliefs about medicines were examined with the BMQ.^8^ The questionnaire has been validated across various illness groups and languages and shown to be stable.^8 25 26^ The questionnaire has previously been translated into Swedish, and the back-translations have been approved by the developer.^27^ Five of the BMQ subscales were used: the subscales measuring Necessity, Concerns, Harm, Overuse and Benefit. Necessity and Concern include five questions each and reflect participants’ beliefs about the medicines that are prescribed for them, while Harm, Overuse and Benefit include four questions each and reflect participants’ beliefs about medicines in general. All questions were answered on a five-point Likert scale (strongly agree=5, agree=4, uncertain=3, disagree=2 and strongly disagree=1), giving sum scores of 5–25 for the subscales Necessity and Concern and 4–20 for the subscales Harm, Overuse and Benefit. A higher sum score gives higher agreement on the subscale.

Data from Riksstroke from the acute care in hospital include type of stroke, level of consciousness at admission, having had a previous stroke and whether treated in a stroke unit and dependency in activities of daily living (ADL) before stroke. Data on the number of stroke events during follow-up was also included from the stroke register. Since ADL data from 24 months is not available in the stroke register, questions on ADL were added to the 24 month study questionnaire. Information on ethnicity was not included.

### Statistical analysis

The sample size calculation has been described previously.^15^ Characteristics of participants are presented as frequencies and percentages. MARS scores were dichotomised as in previous studies, with participants with sum scores of 5–22 being classified as non-adherent and participants with scores of 23–25 being classified as adherent.^27 28^ The difference between 3 and 24 months in the number of individuals classified as non-adherent was tested with McNemar’s test. The change in each individual’s MARS score was calculated as the 3 month value minus the 24 month value, and the mean individual change in MARS score was calculated. Differences in MARS and BMQ scores between 3 and 24 months were tested with the Wilcoxon signed-rank test.

The 24 month results for the BMQ subscales are presented as medians and IQR. The Mann–Whitney U test was used to test differences in BMQ between adherent and non-adherent patients. Multivariable logistic regression models were used to test for associations between the BMQ subscales and self-reported non-adherence. Age and BMQ score were included as continuous covariates in the regression models, and each BMQ subscale was included in a separate model. For categorical covariates, sex was included in the models together with the variable previous stroke, which was statistically significantly associated with adherence in univariate models. Male sex and no previous stroke were chosen as reference categories. Individual who had missing answers on MARS or BMQ subscales were not included in analyses of MARS or that specific BMQ subscale. A difference with a p value <0.05 was considered statistically significant. All analyses were performed with version 26 of IBM SPSS Statistics.

## Results

**Figure 1:**
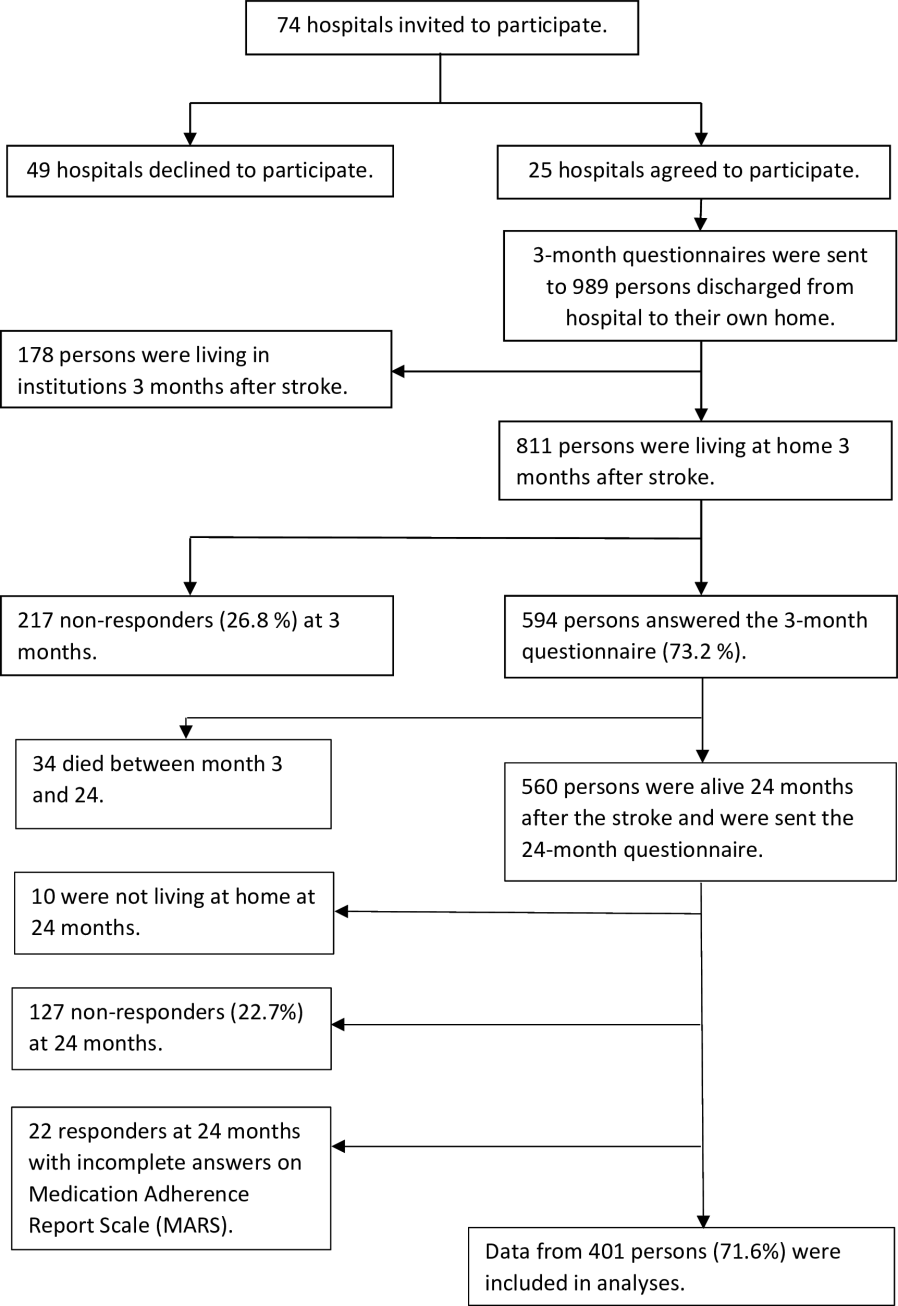
Flowchart of hospitals and participants in the study.

The 3 month questionnaire was answered by 594 individuals who were living at home 3 months after stroke onset. At the 24 month follow-up, 34 (5.7%) had died. The 24 month questionnaire was therefore sent to 560 persons, of whom 127 (22.7%) did not respond and 10 (1.8%) were not living at home, meaning that 423 people were living at home and answered both the 3 month and the 24 month questionnaires. Among these 423 people, 408 and 416 had complete information on MARS at 3 months and 24 months, respectively. Data from the 401 individuals who had complete information on MARS at both 3 and 24 months were used for the analyses, which corresponds to a response rate of 71.6%. Characteristics at stroke onset and after 24 months are presented in table 1, and characteristics at 3 months are presented in online supplemental appendix table 1. Figure 1 shows a flowchart of hospitals and persons in the study.

**Table 1:** Characteristics of study population and comparison between non-adherent and adherent participants at 24 months (n=401)

Variable/characteristic	Non-adherent (n=63)	Adherent (n=338)	p value	Missing cases (n)
Age, n (%)			0.226	0
≤74 years	35 (14.0)	215 (86.0)		
≥75 years	28 (18.5)	123 (81.5)		
Sex, n (%)			0.195	0
Men	44 (17.5)	207 (82.5)		
Women	19 (12.7)	131 (87.3)		
Type of stroke, n (%)			0.881	0
Haemorrhage	5 (16.7)	25 (83.3)		
Other (ICD10 I63+I64)	58 (15.6)	313 (84.4)		
Low level of consciousness at admission, n (%)			0.878	1
No	61 (15.8)	325 (84.2)		
Yes (drowsy or unconscious)	2 (14.3)	12 (85.7)		
History of previous stroke, n (%)			0.049	1
No	49 (14.3)	294 (85.7)		
Yes	14 (24.6)	43 (75.4)		
Treated in stroke unit, n (%)			0.090	1
No	4 (33.3)	8 (66.7)		
Yes	59 (15.2)	329 (84.8)		
Dependent in ADL, n (%)			0.128	1
No	60 (15.2)	335 (84.8)		
Yes	2 (40.0)	3 (60.0)		
***24monthsfollow-up***				
Dependent in ADL, n (%)			0.481	1
No	58 (15.4)	318 (84.6)		
Yes	5 (20.8)	19 (79.2)		
Number of stroke episodes during 2 year period			0.571	5
1	56 (15.2)	312 (84.8)		
2	6 (22.2)	21 (77.8)		
3	0 (0.0)	1 (100.0)		

ADL, activities of daily living

Individuals who did not respond to the 24 month questionnaire (n=127) or who were not living at home (n=10) were more often age over 75 years (p=0.046), women (p=0.046) and dependent in ADL (p=0.042) compared with responders who were living at home.

The number of individuals classified as non-adherent at 24 months (MARS scores 5–22) (n=63, 15.7%) differed significantly (p=0.030) from the number at 3 months (n=45, 11.2%). Of the 356 patients classified as adherent at 3 months, 316 (88.8%) were still adherent at 24 months; and of the 45 patients classified as non-adherent at 3 months, 23 (51.1%) were still non-adherent at 24 months. There was no difference in characteristics between non-adherent individuals who stayed non-adherent in comparison with individuals who changed to adherent (online supplemental appendix table 2). Answers to each of the five MARS questions at 3 and 24 months are given in table 2.

**Table 2:** Answers to each of the Medication Adherence Report Scale questions at 3 and 24 months (n=401)

Individual questions in the MARS questionnaire	Patients self-reporting non-adherent behaviour*, n (%)	
	At 3 months	At 24 months
I forget to take my medicines	37 (9.2)	38 (9.5)
I alter the dose of my medicines	17 (4.2)	19 (4.7)
I stop taking my medicines for a while	7 (1.7)	16 (4.0)
I decide to miss out on a dose	6 (1.5)	11 (2.7)
I take less than instructed	17 (4.2)	23 (5.7)

* The MARS questions have answer alternatives of ‘always’, ‘often’, ‘sometimes’, ‘rarely’, and ‘never’. Non-adherence was defined as an answer of ‘always’, ‘often’, or ‘sometimes’.

There was no significant difference in the mean MARS score between 3 and 24 months (Wilcoxon signed-rank test) (table 3). When comparing the difference in the mean BMQ score between 3 and 24 months, the only significant difference was found in the Necessity subscale, showing that the perceived need for medicines to maintain or improve health increased slightly over time (table 3).

**Table 3:** Differences in Medication Adherence Report Scale and Beliefs about Medicines Questionnaire (BMQ) scores between 3 and 24 months (n=401)

	Scale	Valid cases (n)	Change between 3 and 24 months, mean (SD)	p value*
MARS	5–25	401	0.23 (2.35)	0.139
BMQ Necessity	5–25	379	0.47 (3.08)	0.007
BMQ Concern	5–25	378	−0.30 (4.11)	0.090
BMQ Overuse	4–20	380	−0.21 (2.56)	0.153
BMQ Harm	4–20	375	−0.25 (2.67)	0.113
BMQ Benefit	4–20	380	−0.12 (2.43)	0.498

* Wilcoxon signed-rank test.

Results for the 24 month BMQ subscales are presented in table 4. In the 24 month comparison between non-adherent and adherent people, non-adherent patients scored significantly higher on negative beliefs, *Concern*, *Overuse* and *Harm*, and significantly lower on positive beliefs, *Necessity* and *Benefit*. In the multivariable logistic regression model controlled for age, sex, previous stroke, being treated in a stroke unit, being dependent on help from a relative and memory problems, significant associations were found between all five subscales of the BMQ and non-adherence to treatment. Non-adherent patients had higher scores on negative beliefs about medicines, *Concern* (OR: 1.17, 95% CI: 1.09 to 1.25), *Overuse* (OR: 1.37, 95% CI: 1.21 to 1.54) and *Harm* (OR: 1.24, 95% CI: 1.11 to 1.39), and lower scores on positive beliefs about medicines, *Necessity* (OR: 0.88, 95% CI: 0.80 to 0.96) and *Benefit* (OR: 0.85, 95% CI: 0.74 to 0.98).

**Table 4:** Associations between Beliefs about Medicines Questionnaire (BMQ) subscale scores and non-adherence at 24 months (n=401)

Variable	Valid cases	Scale score median(IQR)		Mann–Whitney U test	Adjusted in multivariable logistic regression models	
		Non-adherent n=63	Adherent n=338	(p value)	(p value)	OR* (95 % CI)
BMQ- Specific						
Necessity	379	17.5 (15–20)	18 (17–21)	0.024	0.003	0.88 (0.80 to 0.96)
Concern	378	15 (12–18)	11 (9–15)	<0.001	<0.001	1.17 (1.09 to 1.25)
BMQ-General						
Overuse	380	13 (12–14)	11 (9–13)	<0.001	<0.001	1.37 (1.21 to 1.54)
Harm	375	11 (10–13)	10 (8–12)	<0.001	<0.001	1.24 (1.11 to 1.39)
Benefit	380	16 (15–17.5)	17 (15–18)	0.023	0.023	0.85 (0.74 to 0.98)

* Adjusted for age, sex, previous stroke, and each BMQ subscale. BMQ, ; OR, odds ratio; CI, confidence interval; , interquartile range. Non-adherent cases were defined as MARS sum score 5–22.

## Discussion

This study, based on a sample of stroke patients in Sweden, showed that between 3 and 24 months after stroke, the beliefs about medicines remained stable across all domains, except for an increased perception of medications as being necessary. The proportion of individuals classified as non-adherent increased. At 24 months after stroke, all subscales of beliefs were still associated with non-adherence.

### Medication adherence

Self-rated adherence in this study seemed to be of the same order of magnitude as in previous studies.^15 16 27^ A meta-analysis on medication adherence after stroke estimated self-reported adherence at 77.7% (95% CI: 70.4 to 84.0).^29^ Including only respondents who were still living at home might have contributed to the slightly higher number in the present study, as less disability and better health have been shown to be predictive of higher medication adherence.^30 31^ The comparative literature on longitudinal change in self-reported adherence is scarce, probably due to methodological challenges. A study by Schüz *et al* with shorter follow-up time reported that both intentional and non-intentional non-adherence remained unchanged after 6 months in older adults with multiple illness, as did a study on older adults with a 2 year follow-up.^32 33^ These results, together with the lack of change in individual MARS scores in the present study, indicate that self-rated medication adherence is quite stable over time even though the proportion classified as non-adherent increased slightly during the longer follow-up.

Most of the adherent individuals at 3 months were also adherent at 24 months; this contrasted with individuals classified as non-adherent at 3 months with almost half of were classified as adherent at 24 months. However, the total proportion of non-adherent patients increased over time because of the larger absolute number of adherent patients switching to non-adherence. No explanation for the change in non-adherence was found when comparing the groups and methodological challenges using self-reported adherence could have contributed. Unfortunately, information on external factors at 24 months such as additional help and support or change in health status, which also are likely to impact adherence, were not included in the study. Although as patients switched in both directions in adherence during follow-up, probably due to changes in circumstances not accessible in the present study, more studies are needed, and interventions aimed at more long-term adherence should probably target both adherent and non-adherent individuals as circumstances affecting adherence change over time. With adequate assistance to take medications as instructed, unintentional adherence should be low and unchanged over time. However, the present study showed, that almost one in ten stroke patients are still forgetting to take their medicines 2 years after stroke (table 2), and improvements targeting non-intentional adherence are also clearly needed. There are good examples to learn from, for example, in the Swedish region of Jämtland, a telephone-based nurse-led secondary preventive programme showed that more patients reached treatment targets for risk factors, by also increasing medication adherence.^34^

There is no clear validated cut-off for non-adherence defined by MARS, and the results in this study are presented dichotomised as well as continuous.^16 27^ This allows for comparisons with other studies using the same instrument and sum score for cut-off as well as for the reader to evaluate changes in adherent behaviour over time. In contrast to the finding of increase in proportion of individuals classified as non-adherent, the mean individual change in total MARS score showed no statistically significant change over time. This could be interpreted as meaning that the individual increases and decreases in MARS score balanced each other out, even though the number of patients with MARS score crossing the pre-specified cut-off for being classified as non-adherent sufficiently showed a change in the proportion of patients being classified as non-adherent. In the previously published 3 month follow-up for the same population, sensitivity analyses with MARS scores 21 and 20 as cut-off decreased the number of non-adherent, but the relationship between BMQ and MARS remained, except for score BMQ-Concern and BMQ-Harm.^15^

### Beliefs about medicines

Some previous studies support the stability of beliefs about medicines over time, which is confirmed in the current study, except for an increased perception of necessity.^13 33^ There are also studies showing that concern increases over time and is associated with increased number of medicines.^14^ In a review study by Sheils *et al*, it was demonstrated that beliefs can be influenced by behavioural change techniques, such as providing information on health consequences, social support and problem-solving.^35^ Stroke patients often have sequelae, risk factors and manifestations of vascular diseases that require support and healthcare contacts, and this may have contributed to an increased perception of the necessity of medications during follow-up. In this study, the proportion of dependent in activities of daily living (ADL) did not change between 3 and 24 months, but ADL is a relatively crude measure that does not always reflect the emergence of other diseases or health issues.

### Influence of beliefs about medicines on medication adherence

At 24 months all subscales of BMQ were still associated with self-reported non-adherence, which is in parallel with results from our previously published 3 months follow-up,.^15^ Non-adherent patients still showed higher scores for concern, overuse, and harm and lower scores for necessity and benefit.^4 13^ The associations between beliefs about medicines and adherence found in the present study are also in line with other studies in other settings.^3236^,^38^ Necessity beliefs, which increased in our study, have also been shown to be important for change in intentional medication adherence.^32^ The mechanisms underlying changes in beliefs about medicines and the potential influence on medication adherence among stroke patients, represents an important area for future research. This would allow for the investigation and testing of interventions, such as techniques aimed at behavioural change. Stroke serves as an excellent example of a chronic condition with a risk of progression where preventive medication therapy is essential.^35^ This includes practical support such as follow-up visits, improved information on medications and correct medication lists, which also would improve non-intentional adherence.

### Strength and limitations

Strengths of this study include a consecutive sample representing both rural and urban areas in Sweden with the same individuals answering the questionnaire twice: 3 and 24 months after a stroke. The repeated cross-sectional design made it possible to follow change over time. Other strengths are the high response rate and the use of previously tested questionnaires.

Although the study population represents stroke patients in both rural and urban areas in Sweden, medication adherence and perceptions of medications are culturally influenced and related to the specific population and disease being studied. This means that comparisons between different countries and illnesses should be made with caution. The present study did not include information on access to support or healthcare interventions during follow-up which could have affected beliefs as well as the adherence.

The importance of methodological considerations in adherence research is well known, and direct comparison between studies using different methods and populations is difficult. Self-reported adherence scales are attractive as they are easy to use but has been shown to overestimate adherence. Systematic reviews have shown that there is a great variation in aspect of adherence measured, as well as the objective comparative method for validation.^24^ Self-estimated adherence using MARS has previously shown to overestimate adherence but was also correlated with blood drug concentrations.^39^ In a study by Norberg *et al*, the 3 month self-reported adherence was as high as refill adherence in the present population, although only 70% were adherent according to both methods indicating that different methods measure different entities of adherence.^40^ Whether these results are also valid for 24 months after stroke has to be evaluated in future studies. Another limitation is the repeated cross-sectional design of the study which does not allow for conclusions about causality.

Limitations include the selection of patients participating in this study. The data were drawn from participants who answered both the 3 month and the 24 month questionnaire, and analyses of non-responders at both 3 and 24 months showed that those who did not respond were older and more dependent in ADL. Our previously published analysis on non-responders to the 3 month questionnaire showed similar results.^15^ Overall, this might indicate that those with the poorest health did not respond to one or both questionnaires.

Data were collected during 2012–2014, but as the study was based on repeated cross-sectional design, the results are still contributing to the understanding of beliefs about medicines and adherence in stroke patients over time. In a recent systematic review on the influences of beliefs about medicines on suboptimal medicine use in elderly, most included studies were cross-sectional and only a few were cohort studies. None of the studies included patients with cardiovascular diseases.^37^ Population data from before 2012 were included, indicating a lack of recent studies following changes in beliefs and adherence. In the beginning of the present decade a pandemic targeting mostly elderly people might have affected the beliefs about medicines. However, this would probably have accentuated the results in the present study with increase in the necessity domain.

## Conclusions

Stroke patients‘ beliefs about medicines were associated with adherence, and over time beliefs remained stable across all domains, except for an increased perception of medications as being necessary. Despite this, more patients became non-adherent over time. To counteract non-adherence, interventions targeted to improve intentional adherence as well as non-intentional adherence should be investigated and implemented.

## supplementary material

10.1136/bmjopen-2024-084680 online supplemental appendix 1

## Data Availability

Data are available upon reasonable request.
