# Supplementary material for: Longitudinal changes in self-reported medication adherence and beliefs about post-stroke medicines in Sweden: a repeated cross-sectional study
Source: BMJ Open. 2024 Oct 18;14(10):e084680. doi: 10.1136/bmjopen-2024-084680 (PMC11492948; doi:10.1136/bmjopen-2024-084680)
Supplement: online supplemental appendix 1 [file bmjopen-14-10-s001.pdf]

Appendix 1

Table 1. Characteristics of study population and comparison between non-adherent and adherent participants at 3 months (n=401).

Variable/Characteristic*	***Non-adherent (n=45)***	***Adherent (n=356)***	***P-value***	***Missing cases (n)***
Age, n (%)			0.185	0
- ≤ 74 years	24 (9.6)	226 (90.4)		
- ≥ 75 years	21 (13.9)	130 (86.1)		
Sex, n (%)			0.056	0
- Men	34 (13.5)	217 (86.5)		
- Women	11 (7.3)	139 (92.7)		
Type of stroke, n (%)			0.155	0
- Haemorrhage	1 (3.3)	29 (96.7)		
- Other (ICD10 I63 + I64)	44 (11.9)	327 (88.1)		
Low level of consciousness at admission, n (%)			0.714	1
- No	43 (11.1)	343 (88.9)		
- Yes (Drowsy or unconscious)	2 (14.3)	12 (85.7)		
History of previous stroke, n (%)			0.011	1
- No	33 (9.6)	310 (90.4)		
- Yes	12 (21.1)	45 (78.9)		
Treated in stroke unit, n (%)			<0.001	1
- No	5 (41.7)	7 (58.3)		
- Yes	40 (10.3)	348 (89.7)		
Dependent in ADL, n (%)			0.517	1
- No	43 (10.9)	352 (89.1)		
- Yes	1 (20.0)	4 (80.0)		
3 months follow-up				
Living alone, n (%)			0.532	4
- No	31 (10.4)	266 (89.6)		
- Yes	13 (13.0)	87 (87.0)		
Dependent in ADL, n (%)			0.473	5
- No	40 (10.8)	330 (89.2)		
- Yes	4 (15.4)	22 (84.6)		
Self-reported poor general health, n (%)			0.836	9
- No	39 (11.1)	313 (88.9)		
- Yes	4 (10.0)	36 (90.0)		
Self-reported depression, n (%)			0.923	14
- No	36 (11.2)	286 (88.8)		
- Yes	7 (10.8)	58 (89.2)		
Dependent of help/support from relatives, n (%)			0.045	11

- No	18 (8.2)	201 (91.8)		
- Yes	25 (14.6)	146 (85.4)		
Self-reported difficulties with memory, n (%)			0.090	5
- Never or almost never	18 (10.7)	150 (89.3)		
- Sometimes	18 (9.7)	168 (90.3)		
- Often or constantly	9 (21.4)	33 (78.6)		

*ADL, activities of daily living.

Table 2. Förslag 1: "Characteristics of study population (n=45), comparison between participants non-adherent at 3 months and non-adherent at 24 months (n=23) with participants non-adherent at 3 months and adherent at 24 months (n=22).

Variable/Characteristic*	***Non-adherent at 3 months and non-adherent at 24 months (n=23)***	***Non-adherent at 3 months and adherent at 24 months (n=22)***	***P-value***	***Missing cases (n)***
Age, n (%)			0.873	0
- ≤ 74 years	12 (50.0)	12 (50.0)		
- ≥ 75 years	11 (52.5)	10 (47.6)		
Sex, n (%)			0.666	0
- Men	18 (52.9)	16 (47.1)		
- Women	5 (45.5)	6 (54.5)		
Type of stroke, n (%)			0.301	0
- Haemorrhage	0 (0.0)	1 (100.0)		
- Other (ICD10 I63 + I64)	23 (52.3)	21 (47.7)		
Low level of consciousness at admission, n (%)			0.139	0
- No	23 (53.5)	20 (46.5)		
- Yes (Drowsy or unconscious)	0 (0.0)	2 (100.0)		
History of previous stroke, n (%)			0.559	0
- No	16 (48.5)	17 (51.5)		
- Yes	7 (58.3)	5 (41.7)		
Treated in stroke unit, n (%)			0.598	0
- No	2 (40.0)	3 (60.0)		
- Yes	21 (52.5)	19 (47.5)		
Dependent in ADL, n (%)*			0.312	1
- No	21 (48.8)	22 (51.2)		
- Yes	1 (100.0)	0 (0.0)		

ADL, activities of daily living.
