# Supplementary material for: Longitudinal changes in self-reported medication adherence and beliefs about post-stroke medicines in Sweden: a repeated cross-sectional study
Source: BMJ Open. 2024 Oct 18;14(10):e084680. doi: 10.1136/bmjopen-2024-084680 (PMC11492948; doi:10.1136/bmjopen-2024-084680)
Supplement: Supplementary file 2 [file bmjopen-2024-084680.reviewer_comments.pdf]

PEER REVIEW HISTORY

BMJ Open publishes all reviews undertaken for accepted manuscripts. Reviewers are asked to complete a checklist review form and are provided with free text boxes to elaborate on their assessment. These free text comments are reproduced below.

ARTICLE DETAILS

Title (Provisional)

Longitudinal changes in self-reported medication adherence and beliefs about medicines post stroke in Sweden – a repeated cross-sectional study.

Authors

Sjölander, Maria; Gustafsson, Maria; Holmberg, Henrik; Glader, E-L

VERSION 1 - REVIEW

**Reviewer**	**1**
**Name**	**Rickles, Nathaniel M.**
**Affiliation**	**University of Connecticut School of Pharmacy**
**Date**	**20-Mar-2024**
**COI**	**No competing interests**

See attachment for comments. Good luck!

Changes in self-reported medication adherence and personal beliefs about medicines. A longitudinal questionnaire survey at 3 and 24 months after a stroke.

The present manuscript is a Swedish study involving a longitudinal survey of stroke patients at 3- and 24-months post stroke. The authors examined the relationships between the different subscales of the Beliefs of Medicines Questionnaire (BMQ) and self-reported medication adherence using the MARS. The authors found overall more individuals self-reported being non-adherent at 24 months after stroke than at 3 months after stroke. Authors reported that non-adherent patients scored higher negative beliefs about medicines, and lower positive beliefs about medicine. There was an increase in perceived necessity of medicines over time.

The manuscript is fairly well written but there are few suggestions to make it stronger.

1. Title: Might be helpful if authors shorten. Perhaps change to: “Longitudinal changes in self-reported medication adherence and beliefs about medicines post stroke.”
2. Abstract: Page 2. Lines 41-48. Please state clearly if the results are significant. Readers should not have to be biostatisticians to interpret Odd Ratios within CI. Please use language regarding statistical significance.
3. Abstract: Authors might want to write more plainly what it means that there was a significant increase in perceived necessity. Does it mean that as time moves forward

patients perceived medications as being more necessary? Putting findings in clearer, accessible language for readers is always helpful and best practice.

4. Strengths and Limitations: Page 3, line 10: Do authors really think their sample as “relatively large”? I would caution against this interpretation since approx. 400 patients doesn’t seem large to many researchers and a bit relative to perspectives.
5. Introduction: It would have been useful to provide statistics of stroke specifically in Sweden. What are the adherence rates of medications for stroke in Sweden? Any specificity of the problem as it relates to Sweden might be useful context/perspectives on the authors’ data presented on Swedish patients.
6. Introduction: To clarify, is BMQ a measure to capture intentional beliefs or non-intentional beliefs or perhaps both? After a discussion of the two types of beliefs, it might help readers contextualize the BMQ as a measure of one type of belief. Please clarify.
7. Introduction: Please clarify why non-adherence to drug treatment after stroke increases over time? If not due to changes in beliefs, why? Some perspectives/literature here might be helpful for the reader.
8. Introduction: Page 4, lines 56-57: Why should readers and others care about beliefs of medicine and its association with self-reported non-adherence beyond 3 months? Why is this important? Why would beliefs change over time? Usually, beliefs are stable without interventions. Please state clearly so readers and others will be motivated to read further and understand the importance of the work.
9. Introduction: This reviewer encourages authors to provide a theoretical framework for their work. There is more than sufficient health behavior theory to support their approach. The authors should clearly state their theoretical framework/theories.
10. Methods: Page 6, lines 34-38. Please clarify what the follow-up questionnaire from the national stroke register measures and why include in the 3-month mail out.
11. Methods: Page 6, line 43. Please clarify what living at home means. Independent living or assisted living?
12. Methods: It might be helpful to readers to briefly state the kind of data in the Riksstroke and how such data comes into the register. Such information might help readers appreciate the quality of the data used from the Riksstroke.
13. Methods: Page 6, 11-15. Please clarify the coding of the MARS as done with the BMQ. That is, what is coded as a 1, 2, 3, 4, and 5? Such clarity provided for the BMQ would be welcomed in the MARS description. Please also share any references regarding the validity and reliability information regarding the MARS and state briefly such information.
14. Methods: Page 6, 26-40. Please provide any validity and reliability information and references for the BMQ and subscales. Please also state for each subscale what the scoring means. Does a higher score on each scale mean greater belief in the necessity of medicines in general, specific to the patient, etc? Please clarify so readers understand the BMQ subscale values as it means to the extent of a particular construct.
15. Methods: Page 7, 42-56. Please move lines 49-51 to the right after line 45 to keep all register data together or combine 45 into sentence 49-51. Why include these variables from the register? Were these variables being used as control variables and why? Why the added variables in the 3-month and 24-month follow-up questionnaire (self-reported depression, self-reported health, ADLs, etc.)? All these variables seem not related to hypotheses or study objectives. Please clarify as confusing for readers as is.

16. Statistical analysis: The authors indicate prior studies dichotomized MARS with only 23-25 scores being adherent and the rest non-adherent. This suggests that the MARS as a tool is only discriminating in adherence at the very top of the measure. Has prior research validated this dichotomization of the MARS with objective and other adherence measures? Please clarify and state for readers.
17. Statistical analysis: Given that the MARS only discriminates at the top end of the range of the scale, why use a continuous measure to detect change? Why not measure if the MARS score changes from nonadherent to adherent? What does a continuous measure of change in the MARS really mean? Please clarify.
18. Statistical analysis: The authors should mention the other variables reported in the methods that were collected from the Riksstroke that were used in the analysis. Awkward presentation of the categorical co-variables. Please state clearly what the categorical covariables were and describe clearly what the reference variables. "Man"? For males? What is the "Never/almost never having memory problems" and "no" reference variables for?
19. Statistical analysis: What was the measure used for statistical difference? P-value at what level? Please clarify.
20. Results: Please state what the response rate was for the analysis.
21. Table 1: Please clarify why some of variables do not add up to 401 or exceed 401. State in the footer if certain variables had missing values, etc.
22. Table 1: Please change "Sex" to "Gender" (also change wherever in text as well). Please also note any ethnicity variability. The lack of it should be provided to readers.
23. Table 1: It might be useful to see if there were any significant differences in study groups across background characteristics from those adherent or non-adherent at 3 and 24 months. Please consider such an analysis since adherence is a primary outcome measure.
24. Tables 2-4: Please indicate total sample size in Table 2-4 analyses based on in the title of table (usually in parentheses, n=401 for example).
25. Table 2: Please clarify in footer of Table how non-adherent vs. adherent was determined and based on MARS. Tables need to stand independent of any text descriptions.
26. Table 3: The authors might move the MARS data (Table 2) before the BMQ table since the BMQ table and analysis is based on the MARS data. Readers need to appreciate the MARS data before applying the BMQ and MARS data to what is currently Table 2. Thus, if done this way, the text needs to move around accordingly.
27. Discussion: The authors might want to summarize in the first paragraph their main findings as it relates to their study objectives and then to a deeper dive into the meaning of the findings in the order of their objectives: stability of beliefs and adherence across study period and then association with beliefs and adherence.
28. Discussion: The discussion of strengths and limitations might be moved to the end of the discussion as with most manuscripts. It is awkward so early in the manuscript and before the discussion of the findings.
29. Discussion: Additional limitations to be noted include: (1) self-reported adherence measures are subject to respondent biases and ideal if findings confirmed with objective measures (could be added to a last section/conclusion on future research directions; and (2) generalizability of findings are limited to Sweden and may not relate to other cultures as we know beliefs are influenced by culture.
30. Discussion: Please remove all pronouns of "we" and "ours" and state text with more objectivity.

31. Discussion: Why do authors believe their results from over 10 years ago is still valid? The pandemic has been a significant disrupter since then and belief systems may have changed especially among older adults who may be more fearful of morbidity and mortality given seeing peers around the world dying from the virus. Please justify why the age of dataset not an issue.
32. Discussion: The authors argue on Pg 14 (lines 8-14) that it made sense that perceived necessity would increase over time given the passing of two years since respondents would be more limited physically. These comments are not consistent with the lack of significant change in ADLs from 3 to 24 months in the sample. The respondents do not seem more disabled and physically limited based on the passage of time. Please clarify.
33. Discussion: Pg 14 (lines 24-26). Authors indicate that intentional non-adherence may still have an impact on long-term drug treatment. How do we know that non-adherence doesn't affect beliefs vs. beliefs affect adherence? The current study is cross-sectional and retrospective such that causality can't be inferred. Please identify cross-sectional and retrospective nature of study as another set of limitations (please add to limitation section).
34. Discussion: Pg 14 (lines 56-58). The authors bring up the concept of medication persistence for the first time in the latter part of the discussion. This reviewer has trouble following their discussion of why adherence and persistence should be considered as separate entities of drug use behavior. How does refill adherence discussion lead to persistence vs. self-report adherence methods comment? The flow and rationale is confusing. Refill adherence and persistence are two separate and not necessarily related concepts. Persistence is how long one remains on the drug. One can have self-reported persistence and adherence and the same with objective measures of persistence and adherence. Frankly, the entire discussion of persistence seems out of place in the discussion of findings and could be reasonably eliminated. The current conversation could be distracting to readers since persistence was not assessed in the study presented.
35. Discussion: Pg 15, lines 30-35. The authors might offer suggestions why almost half of those initially non-adherent became adherence. Is there any sub-analyses that could be done to shed light on the characteristics of these positive switchers that could be the basis for future research, etc?
36. Discussion: The authors bring up the concept of adherence interventions and the need for further improvements. A limitation of their analysis (should be added to limitation section) is that authors do not know what interventions respondents were exposed to during the study. Were those living near an academic teaching facility more likely to have better adherence or switch to adherent behavior than those further away in more distant locations. Such geographical and systems analyses might be a fruitful next set of future research directions.
37. Discussion: Authors are encouraged as noted previously to provide future research directions based on presented findings.
38. Discussion: Authors might clarify that future adherence interventions should target the maintenance of adherence and reduction of non-adherence. Targeting adherence might be awkward vs. maintaining adherence and reducing non-adherence. Not a major concern but maybe a useful distinction.

As many comments above, the manuscript overall has potential and is a useful addition to the literature. Some of the suggestions above are quite minor. Others are more substantive and

require additional content and analyses. The authors are wished the very best in their endeavors to publish this work. Thank you for the opportunity to review this manuscript.

Reviewer **2**

Name **Eickhoff, Christiane**

Affiliation **ABDA - Federal Union of German Pharmacists Associations, Medicine**

Date **22-Mar-2024**

COI **no competing interests**

Dear authors, thank you for this opportunity to review the manuscript. Non-adherence is a major problem across all indications and patient groups and, therefore, a topic that is highly relevant. Especially, data on long-term adherence is scarce, adding to the value of this study. Although the data is quite old already, it is still of interest and adds to the knowledge on this topic in this patient group.

I have no specific suggestions to make with respect to the relatively straightforward methodology used by the authors.

I have the following suggestions for the discussion and conclusion section:

- I suggest to shift the paragraph on the strenght of the study, now located in the second paragraph of the discussion, to the end of the discussion section.

- Page 15: you refer to a paper from Schüz et al stating the their findings are not in line with yours. Could you discuss possible explantations for the differences observed?

- In the limitations of the study you listed the general challenge to measure adherence with self reported measures. Later, you discuss the observed overall increase in non-adherence in the group you studied with a large proportion of patients switching between non-adherence and adherence. This might be due to the methodological challenge. Please include a short commend on this in you discussion section (page 16, line 31to 49).

- You conclude that interventions should be considered for adherent as well as non-adherent patients. Is your intention to state that all patients need interventions on long-term adherence because you cannot diffentiate between them with the tool you used? Or is it that you assume a change in this behaviour?

- Are there any other practical implication you derrive from your results apart from the conclusion that all patients need interventions on adherence?

It is an interesting piece of work, well planned, conducted, and written. Therefore, I recommend the manuscript for publication with minor revision.

VERSION 1 - AUTHOR RESPONSE

- The title has been changed
- We have clarified the conclusion
- The introduction has been expanded and now include the theoretical framework
- We have added more information Beliefs about medicines questionnaire and Medication adherence report scale.
- We have added analyses on patients who switched from non-adherent to adherent.
- Data from 3-months follow-up has been removed and multivariate analyses has been redone without significant changes.
- The disposition of the Discussion section has been changed according to suggestions from reviewers. We also have added a more extensive discussion on limitations and results.

Sincerely

Eva-Lotta Glader
